# Similar clinical outcome after unicompartmental knee arthroplasty using a conventional or accelerated care program: A randomized, controlled study of 40 patients

**DOI:** 10.3109/17453670903035559

**Published:** 2009-06-01

**Authors:** Lotte Borgwardt, Bo Zerahn, Henning Bliddal, Christian Christiansen, Jesper Sylvest, Arne Borgwardt

**Affiliations:** ^1^Department of Orthopaedic Surgery, Frederiksberg University HospitalDenmark; ^2^Department of Clinical Physiology and Nuclear Medicine, Copenhagen University HospitalHerlevDenmark; ^3^The Parker Institute, Frederiksberg University HospitalDenmark; ^4^Department of Anaesthesia, Frederiksberg University HospitalDenmark; ^5^Department of Rheumatology, Frederiksberg University HospitalDenmark

## Abstract

**Background and purpose** Over the last 5 years, there has been increasing interest in reducing length of hospitalization (LOS) through accelerated programs. We examined the clinical outcome of patients undergoing a unicompartmental knee replacement (UKR) in an accelerated care program (A group) compared to a conventional care program (C group).

**Methods** 40 patients randomized into 2 groups were included (A group: 17 patients; C group: 23 patients). Nausea, micturition problems, lower limb dysfunction, pain (VAS), opiate consumption, Knee Society score (KSS), day of discharge, rehospitalization within 3 months, contact with a general physician or nurse, and level of satisfaction were registered. Patients in the A group attended an information meeting. An intraarticular infiltration with Marcaine and adrenaline was used peroperatively. Patients in the C group had an epidural pump for 2 or 3 days. Patients in the A program were treated with NSAID and paracetamol postoperatively. Opiates were used in both groups in the case of breakthrough pain. The patients were considered ready for discharge when they were able to climb stairs to the second floor within 5 min.

**Results** The median length of stay was 1 (1–3) day in the A group and 6 (4–7) days in the C group. The median pain score (VAS) at day 0 was 1 (0–3) in the A group and 5 (0–8) in the C group (p < 0.001). 11/23 of the patients in the C group had weakness of the lower limbs on day 1 due to the epidural; all patients in the A group were exercising on the day of the operation. Micturition problems necessitating intermediate catherization were more frequent in patients in the C program (19/23) than in patients in the A programme (3/17) (p = 0.001). There were no statistically significant differences between the two groups concerning nausea, average pain on days 1 and 2, use of opioids (during the first week postoperatively), KSS, contact with primary sector, level of satisfaction, or level of confidence.

**Interpretation** We achieved a reduction in LOS of 5 days without affecting the clinical outcome.

## Introduction

The length of hospitalization after surgery has decreased considerably over the last 2 decades, including major orthopedic surgery. In 1985 the typical length of stay (LOS) for patients undergoing a unicompartmental knee replacement (UKR) was 15 days (Robertsson et al. 2000) and today it is approximately 5 days (Fisher et al. 1997).

In recent years, there has been an increasing interest in reducing LOS using a fast-track multimodal regime. This regime has mainly been implemented in cardiac surgery (Djaiani et al. 1999), gastrointestinal surgery (Kehlet 2005), and in orthopedic surgery (Husted and Holm 2006, Husted et al. 2006, Larsen et al. 2008). In this randomized, controlled study we compared the clinical outcome of patients undergoing a UKR at the Department of Orthopaedic Surgery, Frederiksberg University Hospital in an accelerated program (A) or in a conventional program (C).

## Patients and methods

Over a 1-year period (February 2004 through January 2005) 402 patients had a knee arthroplasty in our department. 96 of these had a UKR. All patients undergoing a UKR were asked successively to participate in the study, until there were 50 patients for inclusion. The inclusion criteria were: resident in the County of Copenhagen, ASA I or II, no medical history of gastrointestinal bleeding, and someone to look after the patient after discharge. The exclusion criteria were: major psychiatric disease, patients incapable of managing his/her own affairs, inflammatory joint disease, neurological or other disease(s) affecting the lower limbs, and previous major surgery of the knee.

The patients were randomized (using sealed envelopes) into 2 groups, the conventional program group (C) and the accelerated program group (A). The Ethics Committee of the County of Copenhagen (KF 01-231/03) approved the study, and all patients gave informed consent.

The protocol for anesthesia was violated in 3 patients in the A group due to unforeseen events after the randomization, but before the operation. Also, 3 patients scheduled for UKR decided peroperatively to have TKR instead (1 in group A and 2 in group C). 1 patient in group A and 2 patients in group C withdrew their consent regarding participation after randomization, at different stages of the project. Finally, surgery was cancelled for 1 patient in group A because of hypertension. Thus, the study was based on 23 patients in group C and 17 patients in group A. The groups were similar regarding anthropomorphometrical data (Table).

**Table:** Details of patients preoperatively

	Conventional program (n = 23)	Accelerated Program (n = 17)
Age (range)	66 (44–86)	65 (50–81)
Proportion of females	12/23	10/17
Mean BMI of males	29 (22–35)	27 (21–34)
Mean BMI of females	26 (21–45)	29 (20–35)
Mean height of males, cm	178 (167–186)	180 (166–184)
Mean height of females, cm	165 (161–170)	167 (163–175)
Mean weight of males, kg	92 (72–110)	80 (67–115)
Mean weight of females, kg	70 (55–130)	77 (60–105)
Preoperative Knee Society score	35 (20–70)	39 (13–57)
Preoperative function score	60 (30–100)	60 (35–90)

### The perioperative care program

Patients in the A group attended a preoperative information meeting at which an orthopedic nurse, an anesthesiologist, and a physiotherapist thoroughly informed the patients about the planned procedure. Patients in the A group had spinal anesthesia with 3 mL bupivacaine (5 mg/mL) with 5 µg sufentanil added. At the end of surgery, the tissues around the knee joint were infiltrated with 50 mL bupivacaine (2.5 mg/mL) with adrenaline (5 µg/mL).

Patients in the C group were anesthetized using a combined spinal/epidural technique with an indwelling epidural catheter, which was used for continuous infusion with 5 mL/h of bupivacaine (1.25 mg/mL) and morphine (50 µg/mL) for 2 days postoperatively.

All patients had surgery performed by a consultant surgeon. Minimally invasive surgery was used in both programs and all patients were operated without a catheter.

In the A group, postoperative pain was treated with NSAIDs. Opioids were used in both groups in the case of breakthrough pain. All patients were encouraged to walk and were assisted by a trained physiotherapist every day starting on the day of surgery, and this physiotherapist recorded VAS scores.

The patients were considered ready for discharge when they were able to climb stairs to the second floor within 5 min, and this information was given to the patients before the operation. In the A group a 24-hour contact-line was established, and the patients were informed of the opportunity to call for hospital help/assistance after discharge. Furthermore, the personal nurse checked the well-being of the patients by calling them by phone on the day after discharge.

### Outcome measures

The patients were followed according to a strict scheme at 2, 6, 26, and 52 weeks postoperatively. The Knee Society score (Insall et al. 1989) was registered. This is subdivided into a knee score that rates only the knee joint itself and a functional score that rates the patient's ability to walk and climb stairs. It was determined preoperatively and 6 months postoperatively.

The following variables were also registered: nausea, micturition problems (at a level where intermittent catheterization was necessary), lower limb weakness caused by the epidural infusion (patient unable to walk), pain (VAS) at mobilization, consumption of opioids (conversion to morphine using a narcotic conversion table; www.medicin.dk), day of discharge, level of confidence (“how confident did you feel at discharge?”), rehospitalization within 3 months of discharge, phone contact with a general practitioner, contact with a nurse, and level of satisfaction (“how satisfied or dissatisfied were you with the operation and the perioperative period?”) using a Likert scale with 5 categories.

### Statistics

Sample-size calculations were performed with LOS as effect parameter. The expected difference in LOS was 3 days. SDa and the standard deviations (SDa and SDc) were 3 days. With power set to 90% and α set to 0.05, 44 patients would be required. With an estimated dropout rate of 10%, 50 patients would be needed. 10 patients was excluded after randomization. Since they were not different from the rest of their group (by t-test), we decided to use per-protocol analysis of the 40 patients fullfilling the whole program.

All comparisons were performed by parametric or non-parametric tests according to the distribution of data. The level of significance was chosen to be p < 0.05.

## Results

The length of stay was 1 (1–3) day in the A group and 6 (4–7) days in the C group. The median pain score (VAS) for day 0 was 1 (0–2.5) in the A group and 5 (0–7.8) in the C group (p < 0.001); for days 1 and 2 it was similar (median 2).

11/23 of the patients in the C group had weakness of the lower limbs on day 1, due to the epidural infusion preventing them from exercising sufficiently. All patients in the A group had exercise on the day of operation. Micturition problems necessitating catherization were more frequent in patients in the C group (19/23) than in patients in the A group (3/17) (p = 0.001).

There was no statistically significant difference between the groups in the use of morphine during the first postoperative week. Approximately one-half of the patients in both groups experienced nausea during hospitalization.

At discharge, 18 of the 23 of the patients in the C group and 13 of the 17 patients in the A group were “very confident” or “confident”.

After discharge, one-quarter of the patients in both groups had contact with a home nurse. 4/17 patients in the A group and 2/23 in the C group had telephone contact with their general practitioner. No patients were re-admitted to hospital after discharge.

At 6 months postoperatively, the knee score and function score were 93 (35–100) and 90 (35–100) points, respectively, in the C group and 95 (64–100) and 100 (70–100) points in the A group; the differences were not statistically significant. Level of satisfaction was the same in the groups: 11/17 patients in the A group and 14/23 patients in the C group were very satisfied with the operation and with the perioperative period.

## Discussion and conclusion

We accomplished a reduction in LOS of 5 days, from a median of 6 days in the conventional program to 1 day in the accelerated program. This reduction was achieved without affecting the clinical outcome. Furthermore, compared to the conventionally treated group, patients in the accelerated program had fewer problems during rehabilitation and had reduced use of opiates postoperatively. There have been a limited number of studies on accelerated programs concerning hip or knee arthroplasties (Fisher et al. 1997, Swanson et al. 1998, Beard et al. 2002, Isaac et al. 2005, Reilly et al. 2005, Vanhaecht et al. 2005, Ranawat and Ranawat 2007). Only 3 of these have been randomized controlled trials (Swanson et al. 1998, Reilly et al. 2005, Larsen et al. 2008).

Reilly et al. (2005) measured LOS in 41 patients undergoing UKR and found similar results to ours, thus reducing LOS from 4 days in the conventional program to 1 day in their accelerated program. They also found that this reduction in LOS was achieved without impairment of the clinical outcome. It is of interest that despite the wider inclusion criteria we used, we achieved similar results. Reilly et al. set an upper age limit of 75 years and only patients with NSAID tolerance were included, whereas in our study there was no upper age limit and only patients with a medical history of gastrointestinal bleeding were excluded. Furthermore, in contrast to our study Reilly et al. excluded patients with diabetes, previous heart surgery, or deep vein thrombosis.

In the recent study by Larsen et al. (2008), the setup was very similar to the one in our study except that THR, TKR, and UKR were all included. 87 patients were randomized into an accelerated group and a conventional group (THR, n = 56; TKR, n = 27; and UKR, n = 4). LOS was reduced from 8 days in the control group to 5 days in the intervention group. It is difficult to compare the results of that study with ours due to the inclusion of three different patient groups. UKR is a smaller surgical trauma than THR and TKR. It is also well known that the patients undergoing UKR are younger and the LOS expected is therefore shorter. In the study by Larsen et al., one of the discharge criteria was 90 degrees of knee flexion. In our study this degree of flexion was achieved after discharge. Our findings suggest that the degree of knee flexion before discharge has no relevance; we found no difference in knee function scores between the intervention group and the conventional group.

Our accelerated program eliminated lower limb weakness and reduced the number of patients with micturition problems. These 2 improvements are probably due to pain control without epidural infusion. Furthermore, intraarticular infiltration of bupivacaine/adrenaline seems to be a reasonable explanation for the low VAS scores at day 0 in the accelerated programme. This is in accordance with the findings of Ranawat and Ranawat (2007) and of Parvataneni et al. (2007).

The reduction in LOS in our study was achieved without affecting the level of satisfaction or level of confidence, as was also found in 2 other studies (Husted and Holm 2006, Husted et al. 2006). Our findings and those of others support the concept of accelerated postoperative care.

## References

[CIT0001] Beard DJ, Murray DW, Rees JL, Price AJ, Dodd CA (2002). Accelerated recovery for unicompartmental knee replacement—a feasibility study.. Knee.

[CIT0002] Djaiani GN, Cheng DC, Carroll JA, Yudin M, Karski JM (1999). Fast-track cardiac anesthesia in patients with sickle cell abnormalities.. Anesth Analg.

[CIT0003] Fisher DA, Trimble S, Clapp B, Dorsett K (1997). Effect of a patient management system on outcomes of total hip and knee arthroplasty.. Clin Orthop.

[CIT0004] Husted H, Holm G (2006). Fast track in total hip and knee arthroplasty--experiences from Hvidovre University Hospital, Denmark.. Injury.

[CIT0005] Husted H, Hansen HC, Holm G, Bach-Dal C, Rud K, Andersen KL, Kehlet H (2006). Accelerated versus conventional hospital stay in total hip and knee arthroplasty III: patient satisfaction.. Ugeskr Laeger.

[CIT0006] Insall JN, Dorr LD, Scott RD, Scott WN (1989). Rationale of the Knee Society clinical rating system.. Clin Orthop.

[CIT0007] Isaac D, Falode T, Liu P, I'Anson H, Dillow K, Gill P (2005). Accelerated rehabilitation after total knee replacement.. Knee.

[CIT0008] Kehlet H (2005). Fast-track colonic surgery: status and perspectives.. Recent Results Cancer Res.

[CIT0009] Larsen K, Sorensen OG, Hansen TB, Thomsen PB, Soballe K (2008). Accelerated perioperative care and rehabilitation intervention for hip and knee replacement is effective: a randomized clinical trial involving 87 patients with 3 months of follow-up.. Acta Orthop.

[CIT0010] Parvataneni HK, Ranawat AS, Ranawat CS (2007). The use of local periarticular injections in the management of postoperative pain after total hip and knee replacement: a multimodal approach.. Instr Course Lect.

[CIT0011] Ranawat AS, Ranawat CS (2007). Pain management and accelerated rehabilitation for total hip and total knee arthroplasty.. J Arthroplasty (Suppl 3).

[CIT0012] Reilly KA, Beard DJ, Barker KL, Dodd CA, Price AJ, Murray DW (2005). Efficacy of an accelerated recovery protocol for Oxford unicompartmental knee arthroplasty—a randomised controlled trial.. Knee.

[CIT0013] Robertsson O, Dunbar M, Pehrsson T, Knutson K, Lidgren L (2000). Patient satisfaction after knee arthroplasty: a report on 27,372 knees operated on between 1981 and 1995 in Sweden.. Acta Orthop Scand.

[CIT0014] Swanson CE, Day GA, Yelland CE, Broome JR, Massey L, Richardson HR, Dimitri K, Marsh A (1998). The management of elderly patients with femoral fractures. A randomised controlled trial of early intervention versus standard care.. Med J Aust.

[CIT0015] Vanhaecht K, Sermeus W, Tuerlinckx G, Witters I, Vandenneucker H, Bellemans J (2005). Development of a clinical pathway for total knee arthroplasty and the effect on length of stay and in-hospital functional outcome.. Acta Orthop Belg.

